# An intronic VNTR affects splicing of *ABCA7* and increases risk of Alzheimer’s disease

**DOI:** 10.1007/s00401-018-1841-z

**Published:** 2018-03-27

**Authors:** Arne De Roeck, Lena Duchateau, Jasper Van Dongen, Rita Cacace, Maria Bjerke, Tobi Van den Bossche, Patrick Cras, Rik Vandenberghe, Peter P. De Deyn, Sebastiaan Engelborghs, Christine Van Broeckhoven, Kristel Sleegers, On Behalf of the BELNEU Consortium, Johan Goeman, Roeland Crols, Dirk Nuytten, Rudy Mercelis, Mathieu Vandenbulcke, Anne Sieben, Jan L. De Bleecker, Patrick Santens, Jan Versijpt, Alex Michotte, Olivier Deryck, Ludo Vanopdenbosch, Bruno Bergmans, Christiana Willems, Nina De Klippel, Jean Delbeck, Adrian Ivanoiu, Eric Salmon

**Affiliations:** 1 Neurodegenerative Brain Diseases Group, VIB Center for Molecular Neurology, University of Antwerp-CDE, Universiteitsplein 1, 2610 Antwerp, Belgium; 2 Institute Born-Bunge, University of Antwerp, Antwerp, Belgium; 3 Reference Center for Biological Markers of Dementia (BIODEM), Laboratory of Neurochemistry and Behavior, Institute Born-Bunge, University of Antwerp (UAntwerp), Antwerp, Belgium; 4 Department of Neurology, Antwerp University Hospital, Edegem, Belgium; 5 Department of Neurology and Memory Clinic, Hospital Network Antwerp (ZNA) Middelheim and Hoge Beuken, Antwerp, Belgium; 6 Department of Neurosciences, Faculty of Medicine, KU Leuven, Leuven, Belgium; 7 Department of Neurology, University Hospitals Leuven, Leuven, Belgium

**Keywords:** Alzheimer’s disease, ATP-Binding Cassette, Sub-Family A, Member 7 (ABCA7), Variable number tandem repeat (VNTR), Alternative splicing, Cerebrospinal fluid (CSF) biomarkers

## Abstract

Mutations leading to premature termination codons in ATP-Binding Cassette Subfamily A Member 7 (*ABCA7*) are high penetrant risk factors of Alzheimer’s disease (AD). The influence of other genetic variants in *ABCA7* and downstream functional mechanisms, however, is poorly understood. To address this knowledge gap, we investigated tandem repetitive regions in *ABCA7* in a Belgian cohort of 1529 AD patients and control individuals and identified an intronic variable number tandem repeat (VNTR). We observed strong association between VNTR length and a genome-wide associated signal for AD in the *ABCA7* locus. Expanded VNTR alleles were highly enriched in AD patients [odds ratio = 4.5 (1.3–24.2)], and VNTR length inversely correlated with amyloid β_1–42_ in cerebrospinal fluid and *ABCA7* expression. In addition, we identified three novel *ABCA7* alternative splicing events. One isoform in particular—which is formed through exon 19 skipping—lacks the first nucleotide binding domain of ABCA7 and is abundant in brain tissue. We observed a tight correlation between exon 19 skipping and VNTR length. Our findings underline the importance of studying repetitive DNA in complex disorders and expand the contribution of genetic and transcript variation in *ABCA7* to AD.

**Electronic supplementary material:**

The online version of this article (10.1007/s00401-018-1841-z) contains supplementary material, which is available to authorized users.

## Introduction

Alzheimer’s disease (AD) is a highly prevalent, incurable, multifactorial neurodegenerative disease with a long presymptomatic phase of several decades. ATP-Binding Cassette Subfamily A Member 7 (*ABCA7*) was identified as a risk factor for AD through genome-wide association studies (GWAS) [16, 17, 23, 24, 31, 41]. Subsequently, rare heterozygous premature termination codon (PTC) mutations in *ABCA7* were identified and were up to five times more frequent in AD patients. Up to 4% of AD patients in Caucasian populations carry an *ABCA7* PTC mutation [2, 10, 15, 22, 38, 40, 46, 48]. In African Americans (AA) a PTC-causing 44 bp exonic deletion (rs142076058) was identified in 15% of patients, which doubled AD risk, and explained an AA-specific GWAS hit [9]. Risk-increasing PTC mutations are not confined to specific ABCA7 protein domains, proposing reduction of overall *ABCA7* dosage—due to nonsense mediated mRNA decay (NMD) or inactive truncated proteins—as the most plausible pathomechanism. ABCA7 is deemed to be involved in phagocytosis and/or lipid metabolism and is mainly expressed in hippocampal neurons and microglia in the brain [20]. Knockout of the mouse ortholog *Abca7* was shown to reduce phagocytic clearance of amyloid β (Aβ) [12, 21, 39], the main constituent of senile plaques, which together with neurofibrillary tangles and reactive gliosis form the neuropathological hallmarks of AD.

To date, however, a biological variant explaining the GWAS association in Caucasian cohorts remained elusive. None of the PTC mutations are in linkage disequilibrium (LD) with associated GWAS SNPs. Studies have shown association between GWAS SNP rs3764650 and *ABCA7* expression, albeit in opposite directions [3, 49]. Methylation of CpG sites in the *ABCA7* promoter region was independent of this SNP [54]. Possibly, rs3764650 itself could directly affect AD risk [4], however conditional regression analyses suggest a stronger association with a less frequent SNP rs78117248 [10, 22]. No apparent biological consequences can be linked to rs78117248 (e.g. no overlap with transcription factor motifs), but its stronger association suggests the presence of a yet unknown common genetic variant underlying the discovery of *ABCA7* as a susceptibility locus of AD.

We previously sequenced the *ABCA7* locus—including coding, intronic, upstream and downstream sequences—in the Belgian AD cohort, which identified PTC mutations, but did not reveal a functionally strong genetic variant that could explain the GWAS signal [10]. Here we report the identification of a variable number of tandem repeats (VNTR) with a 25 bp repeat unit located in intron 18 of *ABCA7*, immediately flanking exon 18 and including the exon 18 splice donor site (chr19:1049437–1050028 [hg19]). We assessed the role of this *ABCA7* VNTR in AD and associated cerebrospinal fluid (CSF) biomarkers, expression, and alternative splicing.

## Materials and methods

### Study population

Participants were part of a large prospective cohort of Belgian AD patients and healthy elderly control individuals. Patients were ascertained at the Memory Clinic of Middelheim and Hoge Beuken (Hospital Network Antwerp, Belgium), and at the Memory Clinic of the University Hospitals of Leuven, Belgium. Possible, probable, and definite AD diagnosis was based on NINCDS-ADRA [28] and/or NIA-AA [18, 29] criteria. Control individuals were recruited from partners of patients, or were volunteers from the Belgian community. All control individuals scored > 25 on the Montreal Cognitive Assessment (MoCA) test [32], and were negative for subjective memory complaints, neurological or psychiatric antecedents, and family history of neurodegeneration. All participants and/or their legal guardian provided written informed consent before inclusion. The study protocols were approved by the ethics committees of the Antwerp University Hospital and the participating neurological centers at the different hospitals of the BELNEU consortium and by the University of Antwerp. Table S1 provides an overview of individuals for whom DNA, Epstein-Barr virus transformed lymphoblastoid cell lines (LCL), or CSF were included.

### VNTR genotyping

Genomic start and end coordinates of the *ABCA7* VNTR were delineated with the Tandem Repeat Finder algorithm [6], as implemented in the Tandem Repeats Database (TRDB) [14]. To further assess whether the VNTR length could contribute to AD risk, we used the genotyping methods described below.

### ABCA7 VNTR identification with NGS data

We analyzed the depth of NGS sequencing reads aligning to the *ABCA7* VNTR as a proxy for VNTR length. BAM files from 772 AD patients, and 757 matched healthy elderly control individuals—generated as previously described [10]—were used for this purpose. The total number of reads in the BAM file and the number of reads mapping to the VNTR core sequence (chr19:1049514–1049953 [hg19]), which excludes sequencing reads aligning to the VNTR breakpoints, were calculated with Rsamtools [30]. Individuals with very low coverage (≤ 5 VNTR sequencing reads) were not included. VNTR coverage was then normalized by division by the total number of reads for that sample. Association between normalized coverage and SNP genotypes was calculated with a genotypic Kruskal–Wallis test. *D*′ and *r*^2^ LD values were established previously [10].

To analyze the association between VNTR size and GWAS SNP genotypes in an extended population, we used NGS data of the 1000 Genomes Project [1]. Aligned low coverage whole genome sequencing data from European individuals (i.e. British in England and Scotland, Finnish in Finland, Iberian populations in Spain, Utah residents with Northern and Western European ancestry, and Tuscany in Italy) were processed in an analogous manner as the Belgian NGS dataset described above.

### PCR and Sanger sequencing of short VNTR alleles

We designed a PCR protocol to amplify *ABCA7* VNTRs up to approximately 3 kb. This upper limit is determined by PCR efficiency, which is inversely correlated with increasing amplicon size and hampered by high GC content. We used the Kapa 2G Robust HotStart PCR Kit (Sigma-Aldrich, Saint Louis, MO, USA), with a custom protocol as described in the Supplementary Methods. The forward and reverse primer sequences, used in this setting, were respectively 5′-GGCTCAGCCTGGACTTCTAC-3′ and 5′-TCCAAAACCCTGTGATAGCC-3′. The shortest VNTRs were Sanger sequenced, to determine the minimal number of tandem repeats. PCR products were purified with ExoSAP-IT (Thermo Fisher Scientific, Waltham, MA, USA), dideoxy terminated with BigDye Terminator v3.1 Cycle Sequencing Kit (Thermo Fisher Scientific), and processed on an ABI 3730 DNA Analyzer (Thermo Fisher Scientific), according to the manufacturer’s protocol. Analysis was done with SeqMan II software (DNASTAR, Madison, WI, USA).

### Southern blotting

Southern blotting was used to genotype VNTR lengths in 275 patients and 177 control individuals (Table S1, Fig. S1), including individuals without pathogenic mutations [8] in *APP*, *PSEN1*, and *PSEN2*, or *ABCA7* PTC mutations (incl. rs200538373) [10]. Furthermore, we excluded individuals for whom only a single band on Southern blotting was observed, since this could reflect failed detection of the second VNTR allele. The Southern blotting protocol was adapted from the DIG Application Manual For Filter Hybridization (Roche, Basel, Switzerland) (details provided in Supplementary Methods).

Statistical analyses on Southern blotted VNTR alleles were performed in R3.3.2 [35], unless mentioned otherwise. VNTR allele distribution was compared to genotypes of rs3764650 and rs78117248, which were determined previously [10, 45]. Association between SNP genotypes and phenotype was assessed with allelic Fisher exact tests. VNTR length association testing per SNP was done with a Kruskal–Wallis Rank Sum test. Optimal VNTR size cutoff to distinguish expanded and wild-type VNTR lengths was determined as the largest VNTR allele length observed in control individuals without an rs3764650 risk allele. Association testing and calculation of odds ratio (OR) with 95% confidence interval (95% CI) between expanded and wild-type allele carriers in patients and controls was calculated with Fisher exact testing. Calculations of heritability attributable to an *ABCA7* VNTR expansion were performed with INDI-V [52], assuming AD heritability of liability of 79% [13], a 13% risk of AD after age 65 years [42], and a multiplicative model. Association of VNTR length with age at onset (AAO) within patients was calculated with linear regression, using the sum of VNTR alleles or longest VNTR allele, *APOE* ε4 dosage, and gender as independent variables. AAO difference between patients with and without an expanded VNTR allele was assessed using a Mann–Whitney *U* test.

To test whether VNTR allele lengths share a haplotype with *ABCA7* PTC mutations, we used Southern blot to genotype VNTR length in an additional seventeen individuals carrying a previously identified PTC mutation [10]. These individuals were not included in any statistical analyses.

### Correlation between VNTR and CSF biomarkers

CSF concentrations of amyloid β_1–42_ (Aβ_1–42_), phosphorylated tau_181P_ (P-tau_181P_), and total tau (T-tau) were determined for a subset of 168 patients from the Southern blotting cohort (Table S1) as part of their diagnostic work-up with commercially available single parameter ELISA kits (Fujirebio Europe, Ghent, Belgium). Linear regression with the sum of VNTR alleles or longest VNTR allele as the independent variable and log_2_ transformed biomarker levels as the dependent variable was calculated in *R* [35]. Results are reported as *β*-regression coefficients with standard error (SE) and *p* value.

### Expression studies

Procedures for RNA extraction, cDNA generation and characteristics of the LCL cohort are given in Supplementary Methods.

### Quantitative real time PCR

**Fig. 1:**
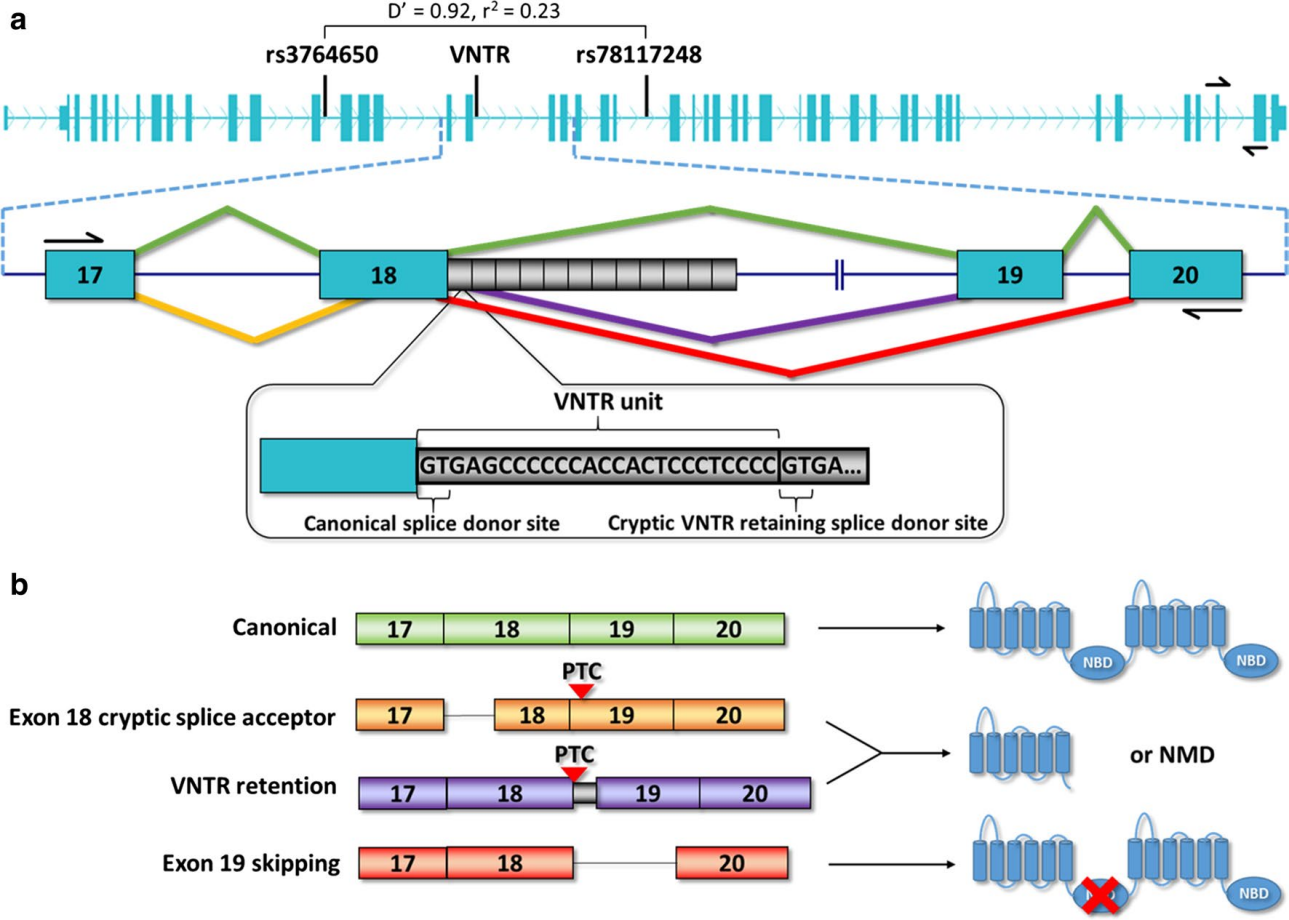
Genomic location of the *ABCA7* VNTR and its effect on *ABCA7* splicing. **a** The *ABCA7* genomic conformation (NM_019112.3) and positions of rs3764650, the VNTR, and rs78117248 are shown. Pairwise *D*′ and *r*^2^ between rs3764650 and rs78117248 is noted on top. Black arrows superimposed on exons 45 and 46 indicate the primers used for qPCR. Below, *ABCA7* exons 17–20 (blue rectangles), introns (black line), VNTR units (black rectangles), and primers in exons 17 and 20 for isoform quantification (black arrows) are represented. Twelve VNTR units are depicted, corresponding to the smallest observed VNTR length. The 25 bp VNTR unit sequence is shown in more detail, with the first two bases corresponding to a splice donor site. Caret-like exon connecting lines depict canonical (green), exon 18 cryptic splice acceptor (orange), retention of a single VNTR unit (purple) and exon 19 skipping (red) splicing. **b** Canonical spicing of the entire *ABCA7* gene leads to a full length ABCA7 protein, containing two nucleotide binding domains (NBD). Usage of a cryptic splice acceptor in exon 18 and retention of a single VNTR unit lead to a frameshift, which causes a premature termination codon (PTC) that leads to either a truncated protein or degradation of the mRNA via nonsense mediated mRNA decay (NMD). Complete skipping of exon 19 causes a deletion of 44 amino acids embedded in the first *ABCA7* NBD

Quantitative real time PCR (qRT-PCR) was performed with the TaqMan Fast Advanced Master Mix (Thermo Fisher Scientific), according to the manufacturer’s protocol in a 384-well plate setting on LCL RNA. Each sample-gene pair was conducted in triplicate and on two different cDNA batches from separate RNA extractions. *ABCA7* transcripts were targeted using the Hs01105117_m1 Taqman assay, with primers located in exon 45 and 46 of *ABCA7* (Fig. 1), and three reference genes were used: *HPRT1* (Hs02800695_m1), *GAPDH* (Hs99999905_m1), and *YWHAZ* (Hs00237047_m1). The PCR protocol (2 min at 50 °C, then 20 s at 95 °C, followed by 40 cycles of 1 s at 95 °C, and 20 s at 60 °C), and fluorescence detection was performed on a ViiA 7 Real-Time PCR System (Thermo Fisher Scientific). Ct values were exported to Qbase+ (Biogazelle, Gent, Belgium) to calculate relative expression.

### Identification of novel alternatively spliced isoforms

Splicing events in proximity to the VNTR were identified using cDNA PCR amplification followed by Sanger sequencing in both LCL and brain cDNA. Forward primers were positioned in exon 17 (5′-TTTCGGAGGAGCTACTGGTG-3′), or on the exon18-cryptic-acceptor-splice-site-breakpoint (5′-GACCCAAAGGCCTGGAGA-3′), and paired with a reverse primer in exon 20 (5′-GTGCTCGTCCACGGTCAG-3′). Amplification was performed with Titanium Taq (Clontech Laboratories, Mountain View, CA, USA) in a 1 M betain solution using the following thermal cycler protocol: 2 min at 95 °C, followed by 35 cycles of 30 s at 95 °C and 1.5 min at 68 °C, and finished by 3 min at 68 °C. The Sanger sequencing reaction was then carried out with BigDye Terminator v3.1 Cycle Sequencing Kit (Thermo Fisher Scientific), and subsequently processed on an ABI 3730 DNA Analyzer (Thermo Fisher Scientific). Analysis was performed with Seqman software (DNASTAR, Madison, WI, USA). All splicing events were further confirmed by analyzing in-house RNAseq data (described in Verheijen et al. [50]) with the use of Integrative Genomics Viewer (IGV) software [37].

### Isoform quantification

Capillary fragment analysis was used to quantify eight observed isoform combinations. PCR amplification of cDNA was analogous to the isoform identification method described above, using a 5′ FAM-labeled forward primer in exon 17 (5′-TTTCGGAGGAGCTACTGGTG-3′), and an exon 20 reverse primer (5′-GTGCTCGTCCACGGTCAG-3′). The PCR amplicons were then supplemented with formamide and GeneScan 600 LIZ Size Standard (Thermo Fisher Scientific) and size separated on an ABI 3730 DNA Analyzer (Thermo Fisher Scientific). Analysis was performed with MAQ-S v1.5.0 software (Agilent). Manual inspection was performed to confirm that fluorescence signals corresponded to the expected size, and signal heights were within quantifiable range. Ratios for canonical, exon 18 cryptic splice acceptor, and exon 19 skipping isoforms were calculated as the signal area of the corresponding isoform peaks divided by the total area of these peaks.

### Allele-specific isoform expression

To examine the effect of individual VNTR alleles on *ABCA7* isoform abundancy we performed allele-specific isoform expression on an individual with a small (837 bp) and large (9711 bp) VNTR allele, and heterozygosis (A/G) for nearby exonic polymorphism rs3752240. VNTR alleles were phased with rs3752240 on genomic DNA according to the “short VNTR allele PCR protocol” (Supplementary Methods) using primers 5′-CAAGACCACCACCCTGTGA-3′ and 5′-AGAGATGGGGAAGGGACCTC-3′. Since the largest allele is too large for PCR amplification, only the shortest allele will be observed, allowing phasing. RNA extraction and cDNA synthesis was performed as described above. Subsequently the “Isoform quantification PCR protocol” was used for which the forward and reverse primers were 5′ supplemented with Illumina Nextera Adapters (5′-TCGTCGGCAGCGTCAGATGTGTATAAGAGACAG-3′ and 5′-GTCTCGTGGGCTCGGAGATGTGTATAAGAGACAG-3′ respectively). The amplicon was then PCR barcoded with i5 and i7 adapters and 300 bp paired end sequenced on MiSeq with v3 chemistry (Illumina, San Diego, CA, USA). GMAP was used to align and output splicing events of sequencing reads [53]. Genotypes for rs3752240 per read were determined with samtools [25].

### Statistical analysis of *ABCA7* overall and isoform quantifications

The qRT-PCR and isoform quantifications were compared to the sum of the VNTR alleles. Since the difference between two VNTR alleles within an LCL sample was kept to a minimum (Supplementary Methods), the VNTR sum value corresponds well to the underlying individual alleles. Association between expression and VNTR sum was calculated with linear mixed-effects models, using lme4 in R [5, 35], correcting for gender and batch in which LCL were grown. The full model was compared to the null model (without VNTR sum) using a Likelihood Ratio test. Linear regression was used to calculate the expression fold change between different VNTR sizes. The analyses were additionally performed using the longest VNTR allele as independent variable.

## Results

### VNTR identification and correlation with the GWAS index SNP at *ABCA7*

We examined repetitive regions in *ABCA7*, which were poorly assessed by conventional NGS data analysis, in our previously generated NGS dataset [10]. This revealed many sequencing reads with low mapping quality in intron 18, close to exon 18, aligning to a low complexity C-rich region. Further inspection indicated a 25 bp tandem repeating pattern. The VNTR comprises 592 bp of the human reference genome (chr19:1049437–1050028 [hg19]), corresponding to 23.7 repetitions of the 25 bp VNTR core repeat motif (Fig. 1). The first two nucleotides (GT) of the VNTR correspond to the canonical splice donor site of exon 18. The sequence between VNTR repeat units can vary slightly (Fig. S2).

First, we investigated the depth of NGS sequencing reads aligning to the VNTR (chr19:1049514–1049953 [hg19]) as a proxy for VNTR length, in relation to the genotype of *ABCA7* GWAS index SNP rs3764650. Carriers of the risk increasing rs3764650[G]-allele had increased read depth in the Belgian cohort (*n* = 1529; Fig. S3, *p* = 2.2 × 10^−16^) [10], as well as in low coverage whole genome sequencing data in European subjects derived from the 1000 Genomes Project (*n* = 403; Fig. S4, *p* = 0.01) [1].

**Fig. 2:**
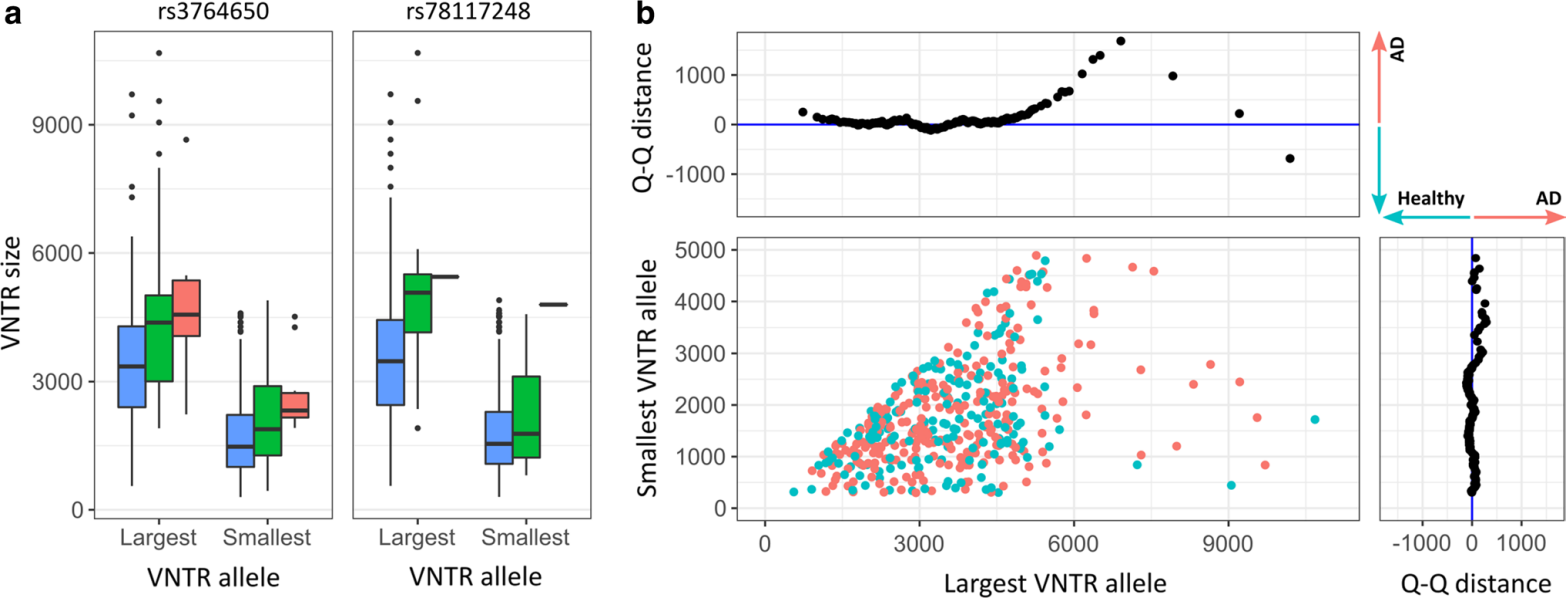
Association of *ABCA7* VNTR lengths with AD and AD-associated SNPs. **a** The distribution of largest and smallest Southern blotted VNTR alleles is shown in relation to the genotype of rs3764650 and rs78117248 in three categories: homozygous reference allele (blue), heterozygous (green), and homozygous risk allele (red) carriers. These distributions are shown per phenotype in Fig. S5. **b** The main panel depicts patients (red) and healthy elderly control individuals (blue) according to their largest (*x*-axis) and smallest (*y*-axis) VNTR allele, as determined by Southern blotting. The upper and right panel show the *Q*–*Q* distance, respectively corresponding to the largest and smallest VNTR allele. A positive distance reflects enrichment in AD patients, while a negative distance corresponds to enrichment in controls

To directly genotype VNTR length, we performed Southern blotting on 452 individuals. For each individual, two size-separated bands were imaged corresponding to the VNTR alleles; no signs of mosaicism were present (Fig. S1). We observed high variability in VNTR lengths between individuals. The smallest allele was 298 bp (approx. 12 repeat units), which could be confirmed with Sanger sequencing. The largest observed allele size was 10678 bp (approx. 427 tandem repeats). Relative to the *ABCA7* VNTR in the human reference genome (chr19:1049437–1050028 [hg19], 592 bp), the longest VNTR length causes an eightfold size increase of intron 18 and a 39% extension of *ABCA7* pre-mRNA. Increasing VNTR lengths were associated with risk alleles of rs3764650 (*p* = 6.7 × 10^−8^) and rs78117248 (*p* = 6.3 × 10^−4^) (Fig. 2a and Fig. S5), confirming our observations based on NGS-based approximation.

We assessed normal variability of VNTR lengths (median = 2388 bp, interquartile range = 2130 bp) within healthy elderly without an rs3764650 risk allele (*n* = 133) and observed a maximum VNTR length of 5720 bp (Fig. S6).

### *ABCA7* VNTR expansion is associated with AD

**Fig. 3:**
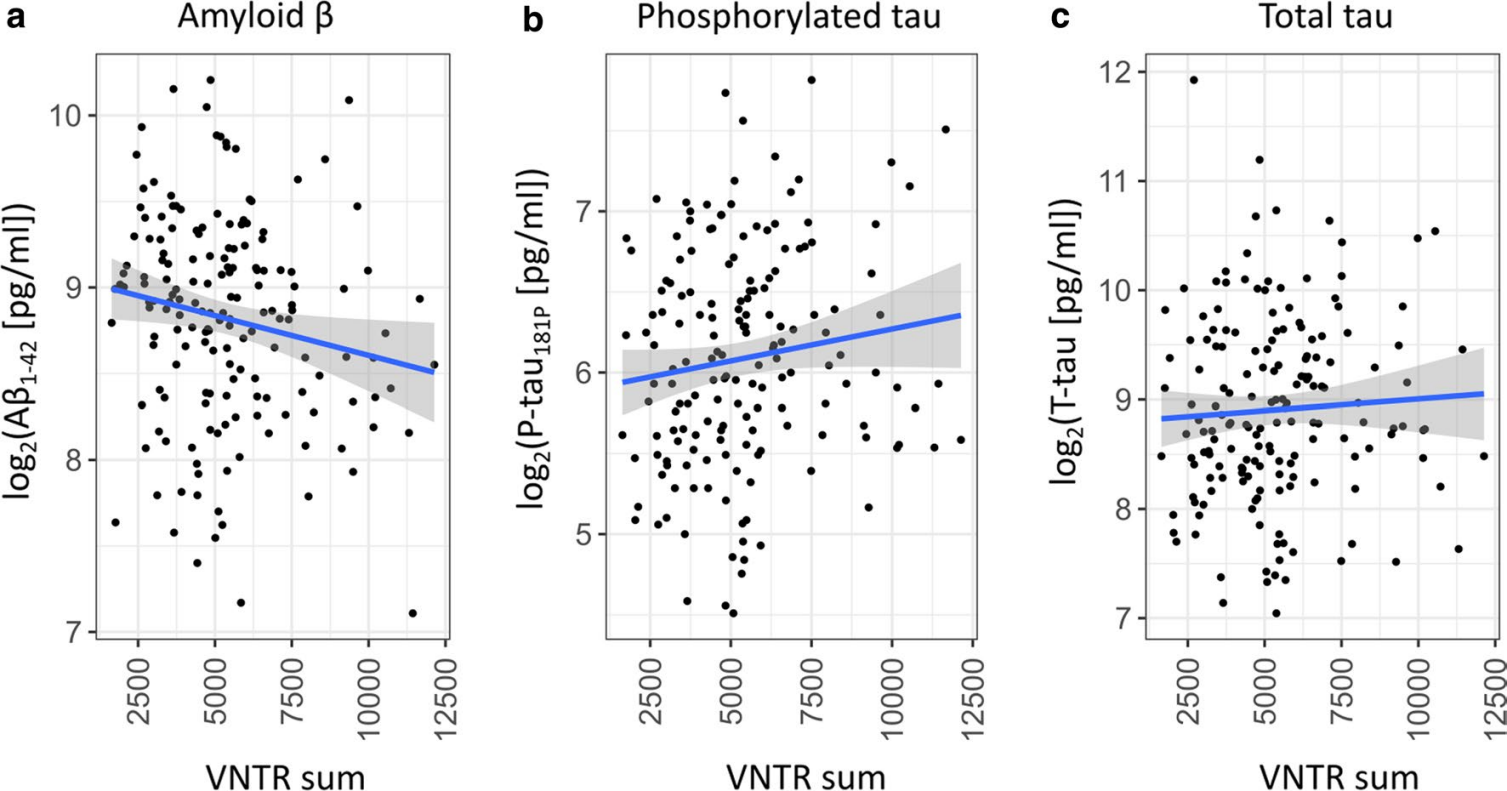
Effect of the *ABCA7* VNTR on CSF biomarkers of AD. Logarithmic levels (*y*-axis) of CSF Aβ_1–42_ (**a**), P-tau_181P_ (**b**), and T-tau (**c**) in AD patients corresponding to the sum of VNTR allele lengths (*x*-axis) within those patients. A trendline (blue) is shown with standard error (shaded area)

For individuals with two alleles within the normal range (< 5720 bp, “wild-type”), distribution was similar between patients and controls (Fig. 2b). However, individuals carrying an allele > 5720 bp (“expanded”), were mostly patients [OR 4.5 (95% CI 1.3–24.2), *p* = 0.008, Fig. 2b]. Twenty patients (7.3%) carried an expanded VNTR allele in contrast to three controls (1.7%) (Table S2). In this cohort, the heritability of liability attributable to *ABCA7* VNTR expansion is estimated at 3.1%. Among carriers of an expanded allele, the second (smaller) allele was longer in patients (2751 ± 1164 bp) than controls (1001 ± 652 bp). In the group with Southern blot data, 25% of patients and 22% of controls carried at least one rs3764650 risk allele. The latter is not significantly associated with AD in this cohort (*p* = 0.6), precluding conditional regression analysis. For expanded VNTR allele carriers, however, the proportion of rs3764650 risk alleles increased to 63 and 100% for patients and controls, respectively (Table S2). We observed no shared haplotype between expanded VNTR alleles and *ABCA7* PTC mutations (Fig. S7). Patients with an expanded VNTR allele had an onset age ranging from 44 to 90 years. The age at inclusion for all three control individuals falls within this range (Table S2). We observed no association between VNTR length and onset age in patients (*p* = 1, Table S3, Table S4), nor for expanded VNTR allele carriers compared to other patients (*p* = 0.9). We analyzed the correlation of VNTR length with CSF biomarkers for AD (Fig. 3a–c) in 168 AD patients. A decrease in Aβ_1–42_ was observed with increasing VNTR length (*β*_log(Aβ)_ = − 4.6 × 10^−5^, SE = 2.1 × 10^−5^, *p* = 0.03 for sum of VNTR alleles). For P-tau_181P_, a directly proportional trend was observed, albeit not significant (*β*_log(p-tau)_ = − 4.0 × 10^−5^, SE = 2.3 × 10^−5^, *p* = 0.09 for sum of VNTR alleles). We did not detect an association of VNTR length on T-tau (Table S3).

### VNTR length is associated with decreased ABCA7 expression

**Fig. 4:**
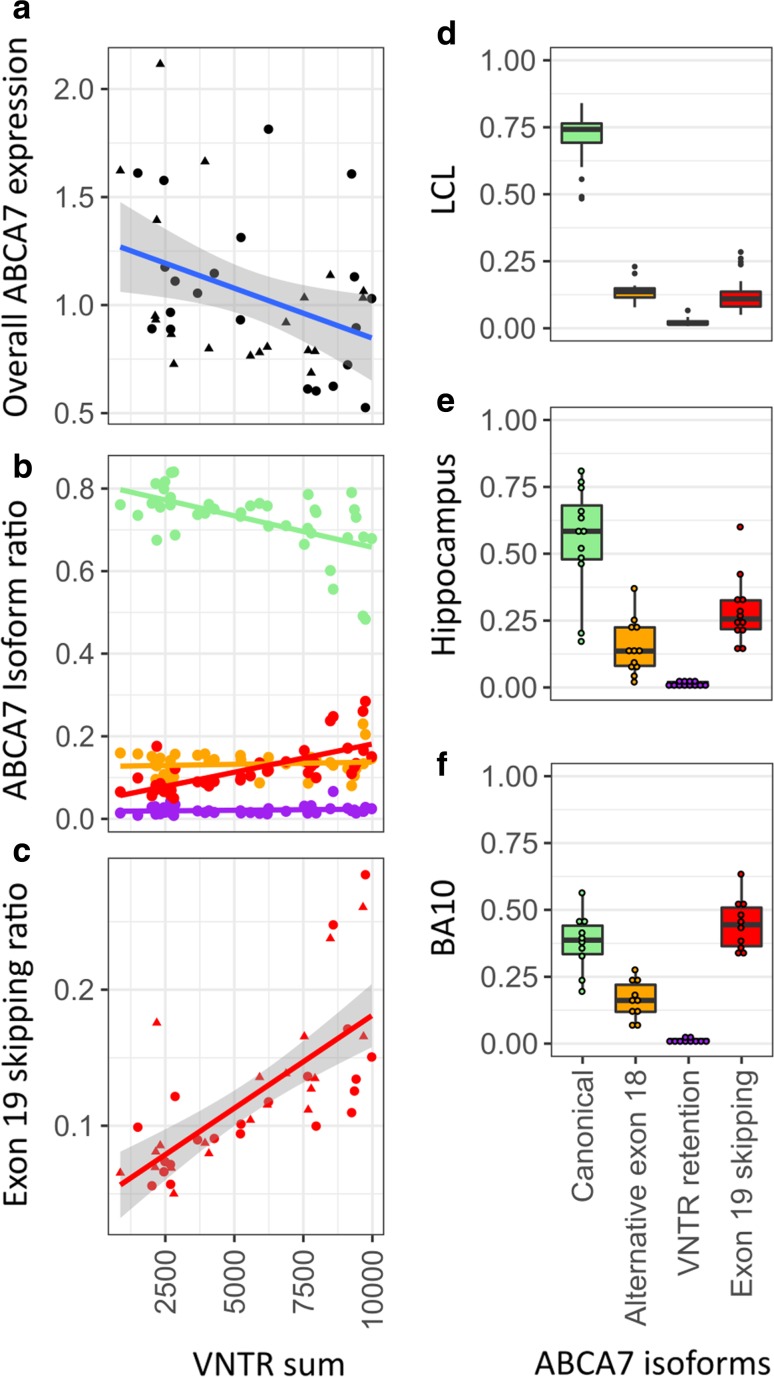
Quantification of *ABCA7* VNTR effects on *ABCA7* expression and splicing. **a**–**c** Quantification of *ABCA7* expression in LCL according to the sum of the two VNTR alleles within an individual (*x*-axis). **a** Relative RT-PCR determined *ABCA7* expression targeting all known isoforms of *ABCA7* for patients (dots) and controls (triangles). A trendline (blue) is shown with standard error (shaded area). **b** The ratio of canonical (green), alternative exon 18 splicing (orange), VNTR unit retention (purple), and exon 19 skipping (red) shown for individuals (dots) and with a trendline per isoform. **c** Exon 19 skipping shown in more detail for patients (dots), and controls (triangles) with a trendline and standard error (shaded area). **d**–**f** Distribution of the four isoforms (*x*-axis) across three different tissues: LCL (**d**), hippocampus (**e**), and Brodmann area (BA) 10 (**f**)

We observed a decrease in overall *ABCA7* expression with increasing VNTR length (*β* = − 4.7 × 10^−5^, SE = 1.9 × 10^−5^, *p* = 0.01 for sum of VNTR alleles) (Fig. 4a, Table S3). This trend was observed for both patients and controls. The average expression for the sample with the largest diploid combination of VNTR alleles (5399 + 4575 bp) was 33% lower than the expression at the smallest diploid VNTR combination (557 + 315 bp).

### *ABCA7* alternative splicing is affected by VNTR length

We observed three separate isoform changes in exon 18 and exon 19 (exons flanking the VNTR), currently unknown to public databases (Fig. 1). These alternative splicing events include usage of a cryptic splice acceptor site in exon 18 (chr19:1049314–1049316 [hg19]), usage of a cryptic splice donor site in intron 18 (chr19:1049461–1049463 [hg19]), and exon 19 skipping. The first two alternative splicing events respectively cause a 52 bp loss of coding sequence, and a 25 bp intron retention (corresponding to one VNTR repeat unit), hence both resulting in an out-of-frame transcript. The third splicing event leads to complete skipping of exon 19, which causes in-frame loss of coding sequence, corresponding to 44 amino acids that are part of the first nucleotide binding domain (NBD) of ABCA7 (Fig. 1). We analyzed the effect of VNTR length on abundance of these splicing events in LCL (Fig. 4b). Exon 18 alternative splicing and intron 18 retention events were not affected by different VNTR sizes (Table S3). Exon 19 skipping, however, strongly increased with expanding VNTR size (*p* = 3.24 × 10^−13^ for sum of VNTR alleles; Table S3). This trend was present in patients and controls (Fig. 4c). Exon 19 skipping was 3.2 times more abundant in the LCL sample with the largest diploid combination of VNTR alleles (5399 + 4575 bp) compared to smallest VNTR combination (557 + 315 bp). To determine the effect of individual alleles on exon 19 skipping, we examined allele-specific expression in LCL of an AD patient with an expanded VNTR allele (9711 bp) and a small wild-type allele (837 bp) (Table S2)—which were respectively phased with the rs3752240[G] and rs3752240[A] allele—and observed a 4.1-fold increase in exon 19 skipping for the expanded allele (Fig. S8).

In addition to quantification in LCL (Fig. 4d), we quantified the three alternative splicing events in the hippocampus, originating from six AD patients and six controls, as well as in Brodmann area 10 (BA10) brain tissue from four patients and six controls (Fig. 4e, f). For each brain tissue, three individuals had VNTR Southern blotted lengths and none carried an expanded VNTR allele. Splicing isoforms causing a frameshift had approximately similar expression level in all tissues: 14 ± 6 and 2 ± 1%, respectively. Exon 19 skipping, however, varied strongly between different brain regions and between brain and LCL: 45 ± 10% in BA10, 29 ± 12% in hippocampus, and 12 ± 6% in LCL. In 90% of BA10 cases and 31% of hippocampal tissue, the cumulative abundance of the alternative splicing transcripts exceeded the abundance of the canonically spliced transcript (Fig. 4e, f).

Since exon 18 alternative splicing and intron 18 retention introduce PTCs, they are subject to NMD. To evaluate whether the VNTR has an effect on NMD efficiency, we analyzed LCL treated with cycloheximide (CHX). Addition of CHX substantially increased the abundance of PTC introducing transcripts (Fig. S9), but no linear relationship was observed between the VNTR sum and the change in transcript abundance (*p* = 0.7 for exon 18 alternative splicing) (Fig. S10).

## Discussion

We report a VNTR in *ABCA7* that increases risk of AD up to 4.5-fold. VNTR length was tightly correlated with a GWAS index SNP for AD in *ABCA7*, with expansions predominantly occurring on the risk allele haplotype. VNTR length was negatively correlated with ABCA7 expression, in line with the mode of action of rare PTC mutations in *ABCA7* that increase risk of AD [2, 9, 10, 15, 22, 38, 40, 46, 48]. Interestingly, VNTR expansion resulted in an increase in exon 19 skipping, a splicing event that has not previously been reported. This splicing event was most prominent in brain tissue.

A genetic variant with downstream biological consequences underlying the GWAS association in *ABCA7* in Caucasian AD cohorts had not been identified yet. Here, we show that individuals with the risk haplotype of rs3764650 and rs78117248 have longer VNTR lengths on average. Furthermore, the majority of carriers with an expanded VNTR allele have at least one rs3764650 risk allele. Within this study population, rs3764650 is not associated with AD due to a comparable risk allele frequency in patients (MAF = 13.4%) and controls (MAF = 12.1%), therefore restricting the use of conditional regression. Nevertheless, the number of patients with an expanded VNTR allele (7.3%) is much higher than controls (1.7%), rendering this *ABCA7* VNTR a common high-penetrant [OR 4.5 (95% CI 1.3–24.2)] risk factor for AD. Expanded VNTR alleles were not in LD with *ABCA7* PTC mutations, and independently contribute to AD risk. Within this discovery cohort, the heritability of liability attributable to expanded *ABCA7* VNTRs equals 3.1%. Compared to other genetic AD risk factors, this estimate is second to only *APOE* ε4 [11].

Increasing VNTR length was associated with an overall reduction of *ABCA7* expression. This points towards a similar pathomechanism as observed in *ABCA7* PTC mutation carriers. We observed no effect of VNTR length on NMD, hence *ABCA7* expression reduction is not caused by differential proportions of alternatively spliced exon 18 and VNTR retention transcripts. The sequence of the *ABCA7* VNTR contains recurring transcription factor binding motifs for ZNF263, PLAG1, and RREB1 [27]. ZNF263 has the strongest expression in brain (GTEx Consortium, accessed November 2017) and contains a Krüppel associated box (KRAB) domain which is involved in transcriptional repression [26]. Hence, increased binding of ZNF263 to longer VNTRs could decrease *ABCA7* expression. Other factors contributing to the effect of the VNTR on ABCA7 expression could be chromatin state in the VNTR region, and stability of pre-mRNA with varying VNTR lengths, warranting further investigation.

VNTR expansion resulted in increased skipping of exon 19, which contains 132 highly conserved nucleotides (55% GERP-score > 2) and encodes 44 amino acids that are part of the first nucleotide binding domain of ABCA7. This domain is required for ATP hydrolysis and export of phospholipids [34]. Skipping of this exon can therefore cause reduction of functional ABCA7. Due to high abundance of this isoform in hippocampus and frontal cortex, it has a strong impact on ABCA7 dosage in brain tissue. Analysis of the VNTR repeat unit sequence with SpliceAid [33] revealed binding motifs for three splice factors in particular: SRp30c, YB-1, and Nova-1. SRp30c—in conjunction with YB-1 [36]—represses the use of a downstream splice acceptor site [44], and was shown to regulate exon skipping in *MAPT*, which is associated with frontotemporal dementia [51]. Increase of SRp30c binding motifs due to *ABCA7* VNTR length could therefore influence skipping of downstream exon 19. Nova-1 is only expressed in neurons where it affects alternative splicing [19] and could potentially explain higher exon 19 skipping levels in brain tissue.

AD patients carrying an *ABCA7* PTC mutation display a wide variability in onset age (45–90 years) [7, 10, 15, 38], which may in part be explained by reduced penetrance due to variation in NMD efficiency and alternative splicing events [38]. In line with this, AD carriers of an expanded VNTR allele had onset ages ranging from 44 to 90 years. We observed no association between onset age and VNTR length. Of note, the age at inclusion for the three control individuals with an expanded VNTR allele varied from 63 to 79 years, which is still within the onset age range. Therefore, the current disease penetrance estimate of this *ABCA7* VNTR could be underestimated; clinical follow-up of these individuals is warranted. We observed nominal significant association between increasing *ABCA7* VNTR length and decrease of CSF Aβ_1–42_, which is one of the hallmarks of AD [29]. This observation is in line with a previous report showing association between the risk allele of rs3764650 and increased neuritic plaque load [43], and exacerbation of plaque load in *Abca7* knockout mice [12, 21, 39].

Due to technical limitations related to long GC-rich tandem repeats, Southern blotting is currently the only method for size determination of the two VNTR alleles within an individual. Southern blotting is a low-throughput method, however, and requires highly concentrated and high molecular weight DNA, which limited the sample size for whom bi-allelic data were available. Nevertheless, due to the strong effect of VNTR expansion on AD risk, we were able to detect association between expanded VNTR alleles and AD in this study cohort. Furthermore, approximation of VNTR length based on NGS data of our full cohort as well as the 1000 Genomes Project supported the association between VNTR and AD risk. Techniques are emerging for target enrichment of GC-rich regions [47], which may in the near future enable a more in-depth characterization of this VNTR at higher throughput. Outstanding questions that should then be explored include studying the effect of intermediate and smaller VNTR lengths on AD and its endophenotypes, epistasis between the two alleles within an individual, sequence differences between VNTR repeat units, nucleotide modifications, and phenotypic heterogeneity due to varying VNTR length.

In conclusion, we identified high penetrant expanded *ABCA7* VNTR alleles in more than 7% of AD patients which increase abundance of a novel NBD-null *ABCA7* isoform. With this study, we emphasize the need for the analysis of tandem repeats and their biological consequences to resolve indirect GWAS signals. Expanded VNTR alleles as well as PTC mutations in *ABCA7* have a strong contribution to AD and both converge to a disease mechanism involving decreased *ABCA7* expression. Future research will be paramount to study this AD subtype with potential benefit from *ABCA7*-specific therapies for AD.

## Electronic supplementary material

Below is the link to the electronic supplementary material.

Supplementary material 1 (DOCX 2142 kb)
