# Supplementary material for: An intronic VNTR affects splicing of ABCA7 and increases risk of Alzheimer’s disease
Source: Acta Neuropathol. 2018 Mar 27;135(6):827–37. doi: 10.1007/s00401-018-1841-z (PMC5954066; doi:10.1007/s00401-018-1841-z)
Supplement: Supplementary file 1 — Supplementary material 1 (DOCX 2142 kb) [file 401_2018_1841_MOESM1_ESM.docx]

Supplementary data: An intronic VNTR affects splicing of *ABCA7* and increases risk of Alzheimer’s disease

Arne De Roeck^1,2^, Lena Duchateau ^1,2^, Jasper Van Dongen^1,2^, Rita Cacace^1,2^, Maria Bjerke^3^, Tobi Van den Bossche^1,2,4,5^, Patrick Cras^4,5^, Rik Vandenberghe^6,7^, Peter P. De Deyn^2,5^, Sebastiaan Engelborghs^3,5^, Christine Van Broeckhoven^1,2^, and Kristel Sleegers^1,2,*^, on behalf of the BELNEU Consortium^$^

^1^Neurodegenerative Brain Diseases group, Center for Molecular Neurology, VIB, Antwerp, Belgium

^2^Institute Born-Bunge, University of Antwerp, Antwerp, Belgium

^3^Reference Center for Biological Markers of Dementia (BIODEM), Laboratory of Neurochemistry and Behavior, Institute Born-Bunge, University of Antwerp (UAntwerp), Antwerp, Belgium

^4^Department of Neurology, Antwerp University Hospital, Edegem, Belgium

^5^Department of Neurology and Memory Clinic, Hospital Network Antwerp (ZNA) Middelheim and

Hoge Beuken, Antwerp, Belgium.

^6^Department of Neurosciences, Faculty of Medicine, KU Leuven, Leuven, Belgium

^7^Department of Neurology, University Hospitals Leuven, Leuven, Belgium

^$^Belgian Neurology (BELNEU) Consortium

^*^Corresponding author: Prof. Dr. Kristel Sleegers MD PhD

Neurodegenerative Brain Diseases Group; VIB Center for Molecular Neurology

University of Antwerp - CDE

Universiteitsplein 1, B-2610, Antwerp, Belgium

Email: kristel.sleegers@molgen.vib-ua.be

# Supplementary methods

## Short VNTR PCR protocol

*Short VNTR allele PCR reaction mix.*

| **Reagent** | **Stock concentration** | **Volume (µL)** |
| --- | --- | --- |
| gDNA | 20ng/µL | 1 |
| KAPA2G Buffer A | 5X | 4 |
| MgCl_2_ | 25mM | 1.3 |
| Forward Primer | 10µM | 1 |
| Reverse Primer | 10µM | 1 |
| KAPA 2G Robust HotStart | 5U/µL | 0.2 |
| Betain | 5M | 10 |

*Short VNTR allele PCR thermal cycler protocol.*

| **Temperature** | **Time** | **Cycles** |
| --- | --- | --- |
| 95°C | 3’ | 1 |
| 95°C | 30” | 35 |
| 57°C | 30” | 35 |
| 72°C | 2’30” | 35 |
| 72°C | 3’ | 1 |

## Southern blotting

DNA sample preparation

Genomic DNA (gDNA) was extracted from patient and control individual LCL or blood tissue (n=452). Purification was performed with QiAmp DNA blood minikit (Qiagen, Hilden, Germany) according to the manufacturer’s protocol. DNA concentration was measured with Dropsense DropQuant96 (Trinean NV, Ghent, Belgium) and integrity of high molecular DNA was determined using ethidium bromide stained 0.8% agarose gel electrophoresis.

Restriction enzyme digestion

Restriction digestion was performed in a volume of 50µL containing 10µg of gDNA, 2.5µL of 20U/µL BamHI enzyme (New England Biolabs, Ipswich, MA, USA), and 5µL of 10X 3.1 buffer. Reactions were completed overnight at 37°C in a thermal cycler. BamHI was inhibited by addition of 5µL SLM, and concentration in a Concentrator 5301 (Eppendorf, Hamburg, Germany) under vacuum at 60°C, after which the volume was brought to 10µL with water.

Agarose gel electrophoresis

Concentrated digested samples where loaded on a 0.8% TBE agarose gel, submerged in TBE buffer. DIG labeled molecular marker II (Roche) was added in separate lanes. Gel electrophoresis was conducted at 60V for 17 hours. Fragment size distribution was examined by briefly staining gels with ethidium bromide and UV illumination.

To depurinate DNA, agarose gels were submerged in 250mM HCl for 10 minutes. After rinsing with ultra-pure water, denaturation was performed by submersion in 0.5M NaOH, 1.5M NaCl buffer for 30 minutes, followed by an ultra-pure water rinsing step. The gels were then neutralized for 30 minutes in 0.5M Tris HCl, pH7.5, 1.5M NaCl. Finally, the agarose gels were equilibrated for at least 10 minutes in 20x SSC.

DNA transfer

DNA was then transferred from the agarose gel to a positively charged nylon membrane using capillary forces with the following setup from bottom to top: a 20x SSC reservoir, a Whatmann paper (Machery-Nagel, Düren, Germany) bridge, agarose gel, positively charged nylon membrane (Roche), Whatmann paper, and a stack of paper towels. The DNA was then UV crosslinked to the membrane in UV illuminator for 3 minutes, after which the membrane was rinsed in double sterile water and air dried.

DIG labeled probe preparation

DIG-labeled probes were generated through PCR amplification in a total volume of 50µL using the conditions depicted below. gDNA with known sequence at primer hybridization sites was used as template, Platinum Taq reagents were used (Invitrogen, Carlsbad, CA, USA), and the PCR DIG mix containing regular dNTPs as well as DIG-labeled dUTPs were part of the PCR DIG Probe Synthesis Kit (Roche). Forward and reverse primers respectively correspond to 5’-AGCTCTGTAACTGCCAGTGC-3’ and 5’-CCGTAGGCTCGTCCAGGAT-3’ DNA oligonucleotides. PCR amplification resulted in specific amplification of a 667 bp probe.

*DIG labeled probe PCR mix*

| **Reagent** | **Stock concentration** | **Volume (µL)** |
| --- | --- | --- |
| gDNA | 100ng/µL | 1 |
| Platinum Taq polymerase | 50X | 0.5 |
| Platinum Taq PCR-buffer | 10X | 5 |
| MgCl_2_ | 50mM | 2 |
| Forward Primer | 10µM | 2 |
| Reverse Primer | 10µM | 2 |
| PCR DIG mix | 10x | 5 |
| H_2_O |  | 32.5 |

*Thermal cycler protocol for probe DIG labeling*

| **Temperature** | **Time** | **Cycles** |
| --- | --- | --- |
| 95°C | 2’ | 1 |
| 95°C | 30” | 35 |
| 66°C | 30” | 35 |
| 72°C | 1’ | 35 |
| 72°C | 7’ | 1 |

Hybridization

DNA-bound nylon membranes were placed in a Techne hybridization tube (Sigma-Aldrich) and submerged in 20mL DIG Easy Hyb (Roche). The tubes were then rotated for two hours at 52°C. Afterwards, the buffer was replaced with hybridization solution (15mL DIG Easy Hyb with 30µL DIG labeled probe (denatured for 5 minutes at 95°C)) and tubes were rotated for 16 hours at 52°C. Membranes were washed by first incubating twice in 2xSSC + 0.1% SDS for 5 minutes, and then twice in 0.1xSSC + 0.1% SDS for 15 minutes at 68°C.

Imaging

Membranes were prepared for imaging with the DIG Wash and Block Buffer set (Sigma-Aldrich). First, membranes were submerged in Washing Buffer for 2 minutes, followed by incubation in Blocking Buffer for 30 minutes. Then the membranes were submerged in Antibody Solution (20mL Blocking Buffer + 4µL Anti-Digoxigenin-AP (Roche)) for 30 minutes. Membranes were then washed twice with Washing buffer for 15 minutes. Equilibration was performed by submersion in Detection Buffer for 3 minutes. Finally CDP-Star (Sigma-Aldrich) was applied dropwise to the membrane surface, after which imaging was performed on an ImageQuant LAS4000 imager (GE Healthcare Life Sciences). Analysis was then performed with ImageQuant TL (GE Healthcare Life Sciences, Pittsburgh, PA, USA). Lanes and fragments were annotated and inspected manually, after which fragment size scoring was done based on the known fragment sizes of the DIG molecular marker II. According to the hg19 reference sequence, Southern blotting sizing incorporates 2038 bp of VNTR flanking sequence due to BamHI restriction digestion, which is subtracted to obtain the corresponding VNTR sizes.

## RNA extraction and cDNA synthesis

Brain RNA extraction

We examined alternative splicing of *ABCA7* in hippocampal and frontal cortex (Brodmann area 10) fresh frozen brain tissue from individuals of Belgian ethnicity. Hippocampal tissue originated from six AD patients (67% female, mean age at death 81.7 ± 6.9 years, mean disease duration 7.7 ± 4.6 years, and mean post-mortem interval (PMI) 3.7 ± 1.3 hours) and six healthy control individuals (33% female, mean age at death 78.0 ± 8.4y, mean PMI 4.2 ± 1.9h). Three individuals had Southern blotted *ABCA7* VNTR lengths, with the largest allele corresponding to 1777, 1783, and 4185bp. Frontal cortex was obtained from four AD patients (50% female, mean age at death 81.5 ± 4.2y, mean disease duration 7.3 ± 5.3y, mean PMI 3.6 ± 1.2h) and six controls (67% female, mean age at death 78.3 ± 8.4y, mean PMI 4.3 ± 2.1h). Three had *ABCA7* VNTR lengths, with the largest allele length determined as 1783, 3131, and 4192bp. Total RNA was isolated from fresh frozen brain samples stored at -80°C. RNA isolation was performed using 100 mg for each sample with the RNeasy plus universal mini kit (Qiagen) according to manufacturer's protocol.

LCL RNA extraction

To study the overall and isoform specific expression of *ABCA7* in a controllable single cell-type environment, we selected 41 LCL derived from 21 AD patients and 20 healthy controls (Table S1). VNTR Southern blotting data and NGS data were available for all. The difference between the two VNTR allele lengths within an individual was kept to a minimum. For the majority (n = 32, 78%) of LCL this difference was less than 1 kb, with the largest difference being 3077 bp. Due to practical feasibility, LCL were grown in four batches. LCL were cultured with 1640 RPMI medium (Thermo Fisher Scientific), supplemented with 15% Fetal Bovine Serum, 2mM L-Glutamine, 1mM Sodium Pyruvate, 100 IU/mL Penicilline, and Streptavidine. Prior to RNA extraction, cell viability was assessed with trypan blue based counting in a Luna II (Logos Biosystems, South Korea) automated cell counter. Five million living cells were then seeded in 2 flasks, after which, one was supplemented with cycloheximide up to a concentration of 150µg/ml. After 4 hours of incubation, cells were centrifuged at 220g for 7 minutes at 4°C, and the medium was then removed to retain the cell pellets. RNA extraction was performed with RiboPure (Thermo Fisher Scientific) according to the manufacturer’s protocol. Briefly, cell pellets were lysed and homogenized in 1mL TRI Reagent. Then, 200µL of chloroform was added after the samples were centrifuged at 12000g for 10 minutes at 4°C. The RNA containing aqueous phase was extracted, supplemented with ethanol, and then transferred to a Filter Cartridge. After centrifugation at 12000g for 30 seconds, the Filter Cartridges were washed twice with 500µL Wash Solution. Finally, RNA was eluted from the filter cartridge in 100µL of Elution Buffer and stored at -80°C.

RNA purification, and cDNA synthesis

RNA from brain and LCL was DNase treated to remove residual DNA with Turbo DNA-free (Thermo Fisher Scientific). Fifty microliter of RNA was supplemented with 5µL 10x TURBO DNase Buffer and 1µL of TURBO DNase and incubated at 37°C for 30 minutes. The mixture was then resuspended in 5µL DNase Inactivation Reagent and incubated for at least 5 minutes at room temperature. After centrifugation at 2000g for 5 minutes, the RNA containing supernatant was retained.

RNA integrity number (RIN), and concentration were determined with Bioanalyzer RNA 6000 Nano Kit (Agilent Technologies, Santa Clara, CA, USA), according the manufacturer’s protocol. All LCL samples had a RIN value of at least 9. Mean RIN for hippocampus and frontal cortex respectively amounted to 7.1 ± 1.5 and 8.3 ± 1.0.

First-strand cDNA synthesis was carried on with SuperScript III (Invitrogen). First, 8µL of DNase treated RNA was supplemented with 1µL of 10mM dNTPs and 1µL 50ng/µL random hexamers and incubated at 65°C for 5 minutes. Then, 10µL of cDNA Synthesis Mix (2µL 10x RT buffer, 4µL 25mM MgCl_2_, 2µL 0.1M DTT, 1µL RNaseOUT (40 U/µL), and 1µL SuperScript III RT (200U/µL)) was added, and the mixture was incubated for 10 minutes at 25°C, 50 minutes at 50°C, 5 minutes at 85°C, and chilled on ice. Lastly, 1µL of RNase H was added and incubated for 20 minutes at 37°C. The resulting cDNA reaction was then stored at -20°C.

# Supplementary figures

**Fig. S1: Southern blotting of the *ABCA7* VNTR**


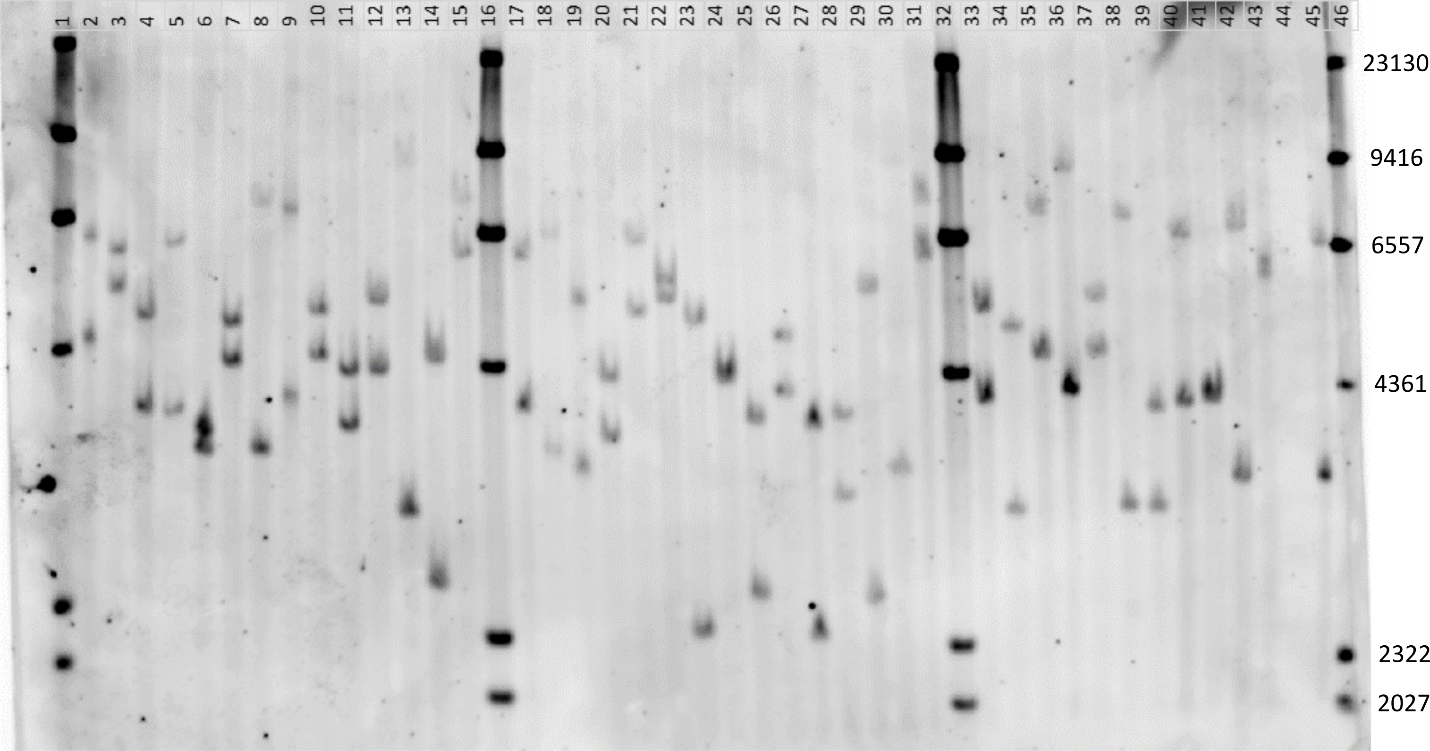


DIG-labeled Southern blotting targeting the *ABCA7* VNTR, with lane numbers shown on top. Lanes 1, 16, 32, 46 correspond to the six largest fragments (base pair length shown on the right) of DIG-labeled DNA molecular weight marker ii, which encompasses the entire VNTR range (of note, *ABCA7* VNTRs have 2038 bp of flanking sequence due to restriction enzyme digests). All other lanes represent separate individuals with bands corresponding to the *ABCA7* VNTR allele sizes.

**Fig. S2: *ABCA7* VNTR tandem repeat motif**


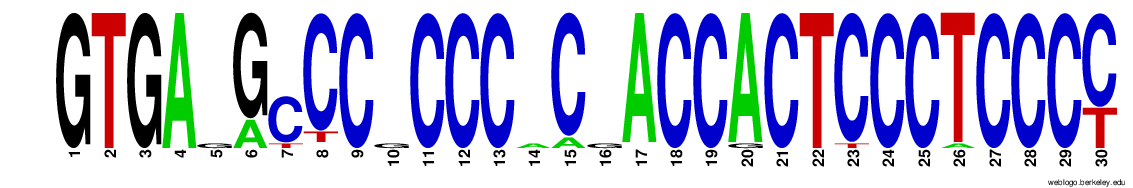


The *ABCA7* VNTR has a 25 bp core repeat unit, with occasional single nucleotide insertions (observed on positions 5, 7, 10, 14, and 16) and base substitutions. The size of each nucleotide corresponds to its frequency on that position according the reference sequence (hg19).

**Fig. S3: Distribution of VNTR NGS sequencing reads in Belgian population according to rs3764650 genotype**


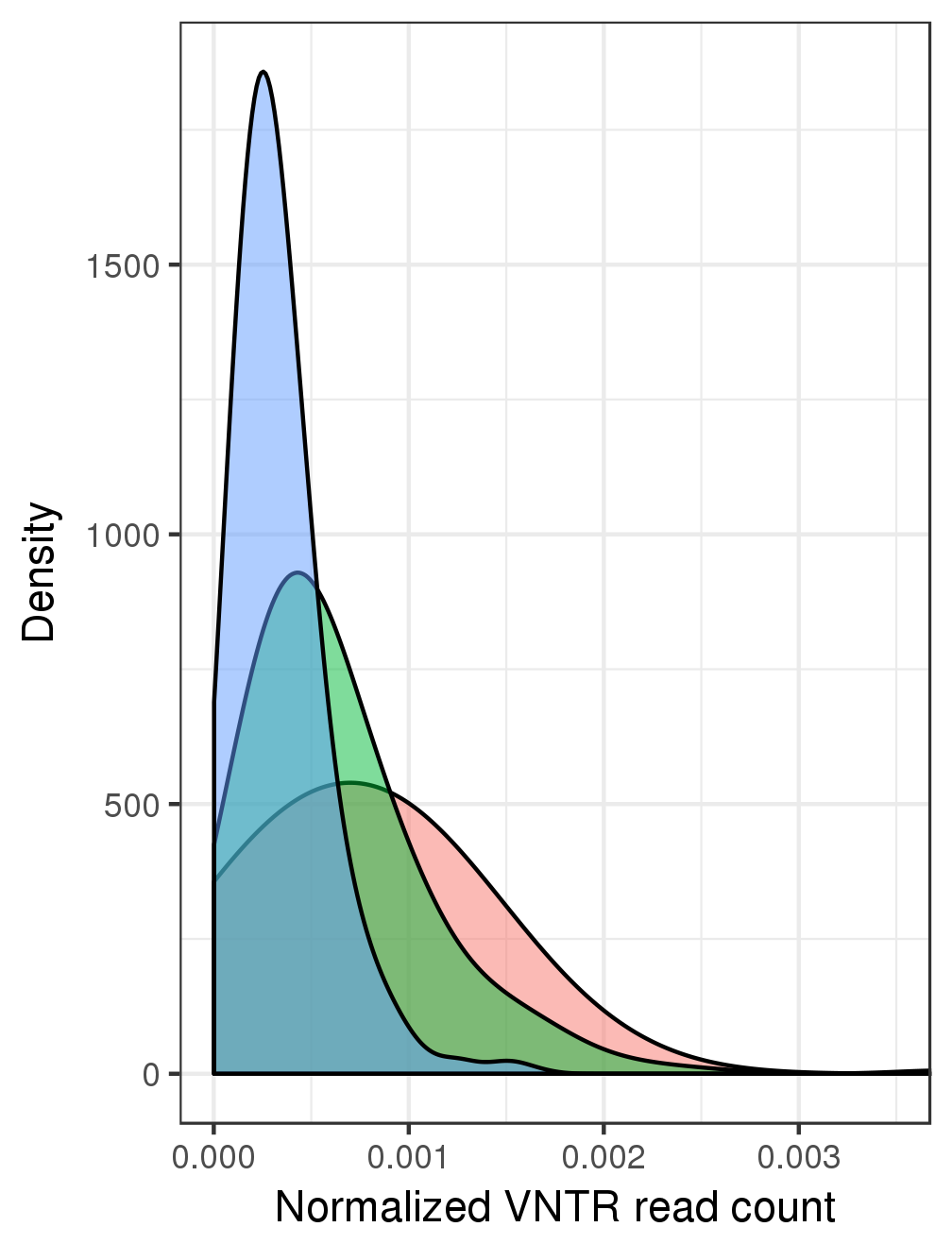


Distribution of normalized sequencing read depth mapping to the *ABCA7* VNTR region according to the genotype of rs3764650. Homozygous major allele carriers (TT) are shown in blue, heterozygous (TG) in green, and homozygous risk allele carriers (GG) in red. Data originated from a previous Belgian cohort NGS study [2].

**Fig. S4: Distribution of VNTR NGS sequencing reads in European 1000 Genomes Project data according to rs3764650 genotype**


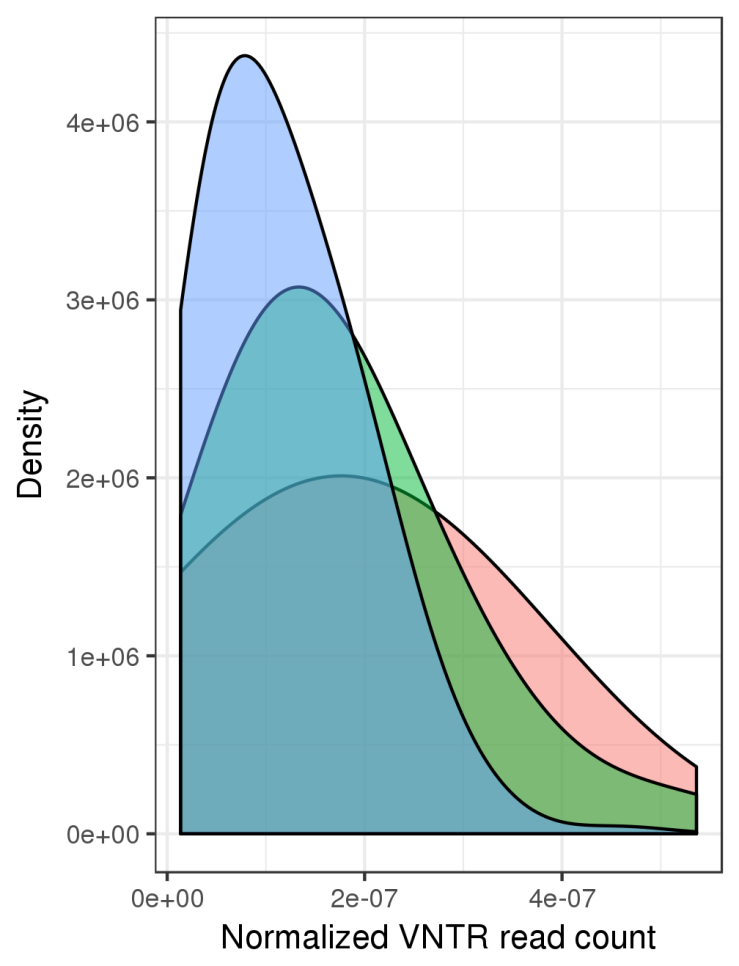


Distribution of normalized sequencing read depth mapping to the *ABCA7* VNTR region according to the genotype of rs3764650. Homozygous major allele carriers (TT) are shown in blue, heterozygous (TG) in green, and homozygous risk allele carriers (GG) in red. Data originated from the European populations in the 1000 Genomes Project [1].

**Fig. S5: VNTR length - GWAS SNP distributions separated by phenotype.**

| **a rs3764650 in controls** | **b rs3764650 in patients** |
| --- | --- |
| 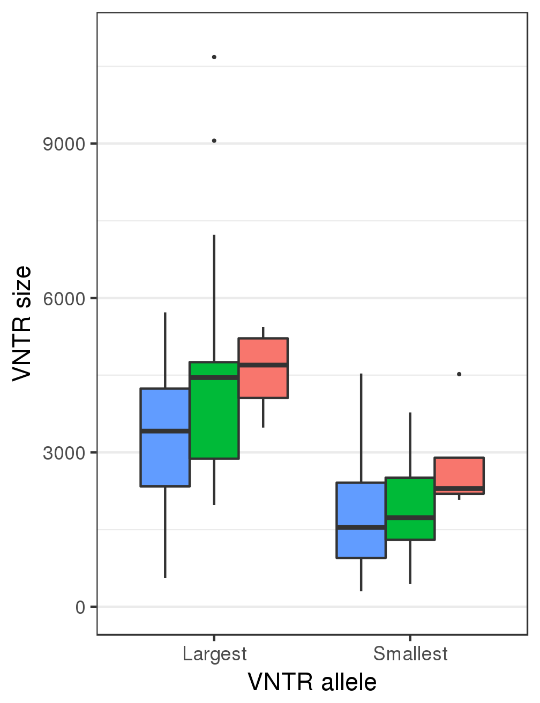 | 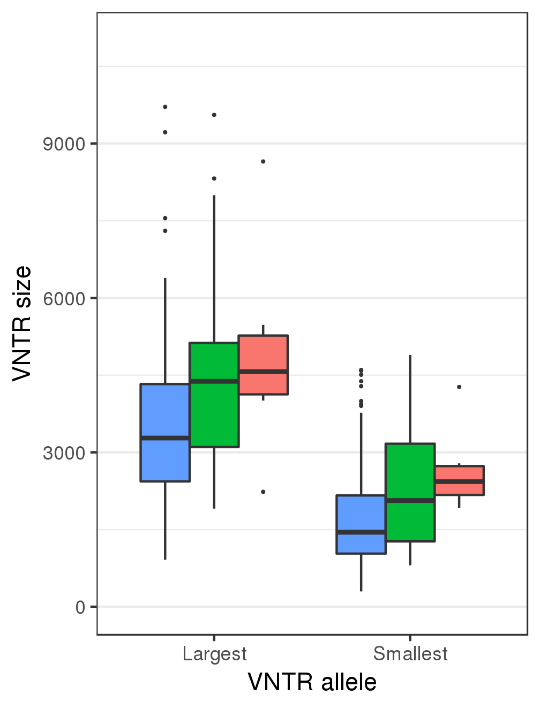 |
| **c rs78117248 in controls** | **d rs78117248 in controls** |
| **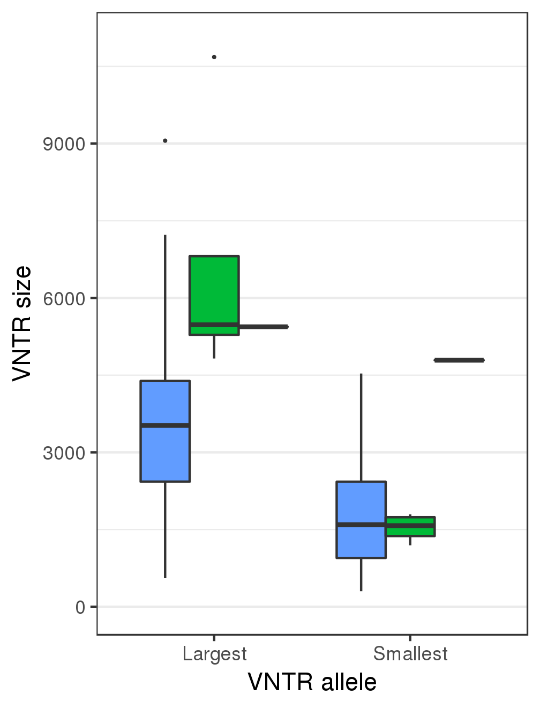** | **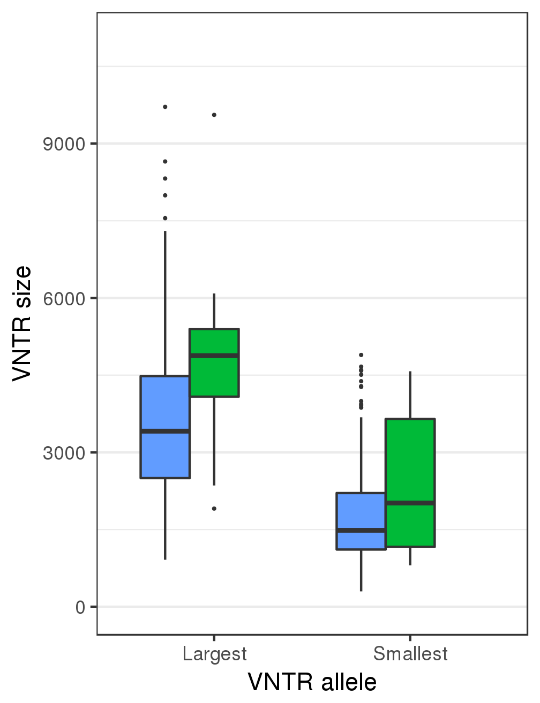** |

A more detailed representation of Fig. 2a, split per phenotype: controls **(a and c)** and AD patients **(b and d)**. The distribution of largest and smallest Southern blotted VNTR alleles is shown in relation to the genotype of rs3764650 **(a and b)** and rs78117248 **(c and d**) in three categories: homozygous reference allele (blue), heterozygous (green), and homozygous risk allele (red) carriers.

**Fig. S6: Distribution of wild-type *ABCA7* VNTR alleles**

| **a** | **b** |
| --- | --- |
| 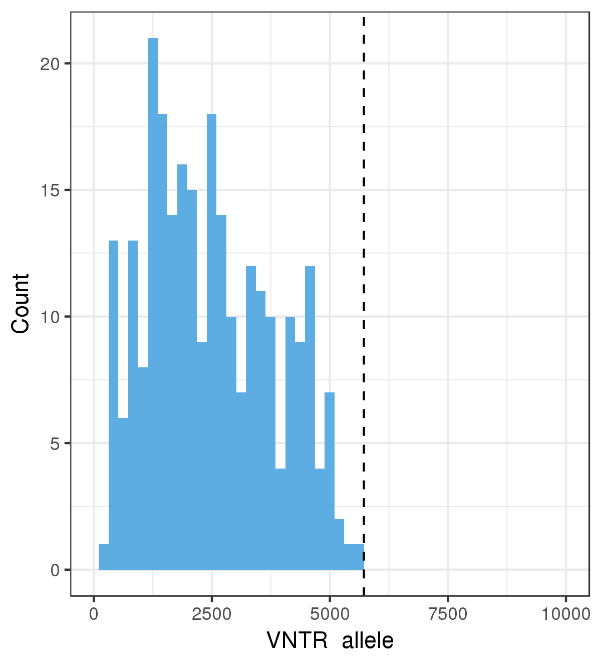 | 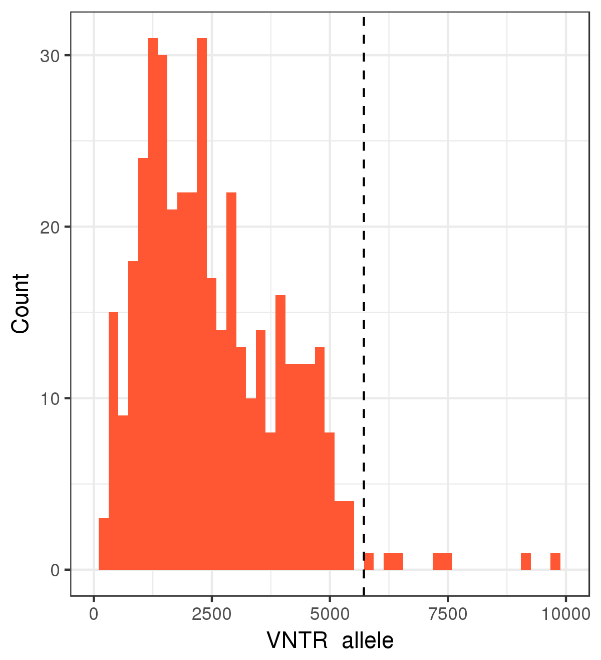 |

Histograms showing the distribution of *ABCA7* VNTR alleles in the healthy elderly control population without an rs3764650 risk allele **(a)** and in the patient population without an rs3764650 risk allele **(b)**. All VNTR lengths in healthy individuals are smaller than 5720 bp (dotted line).

**Fig. S7: VNTR lengths in carriers of *ABCA7* PTC carriers**


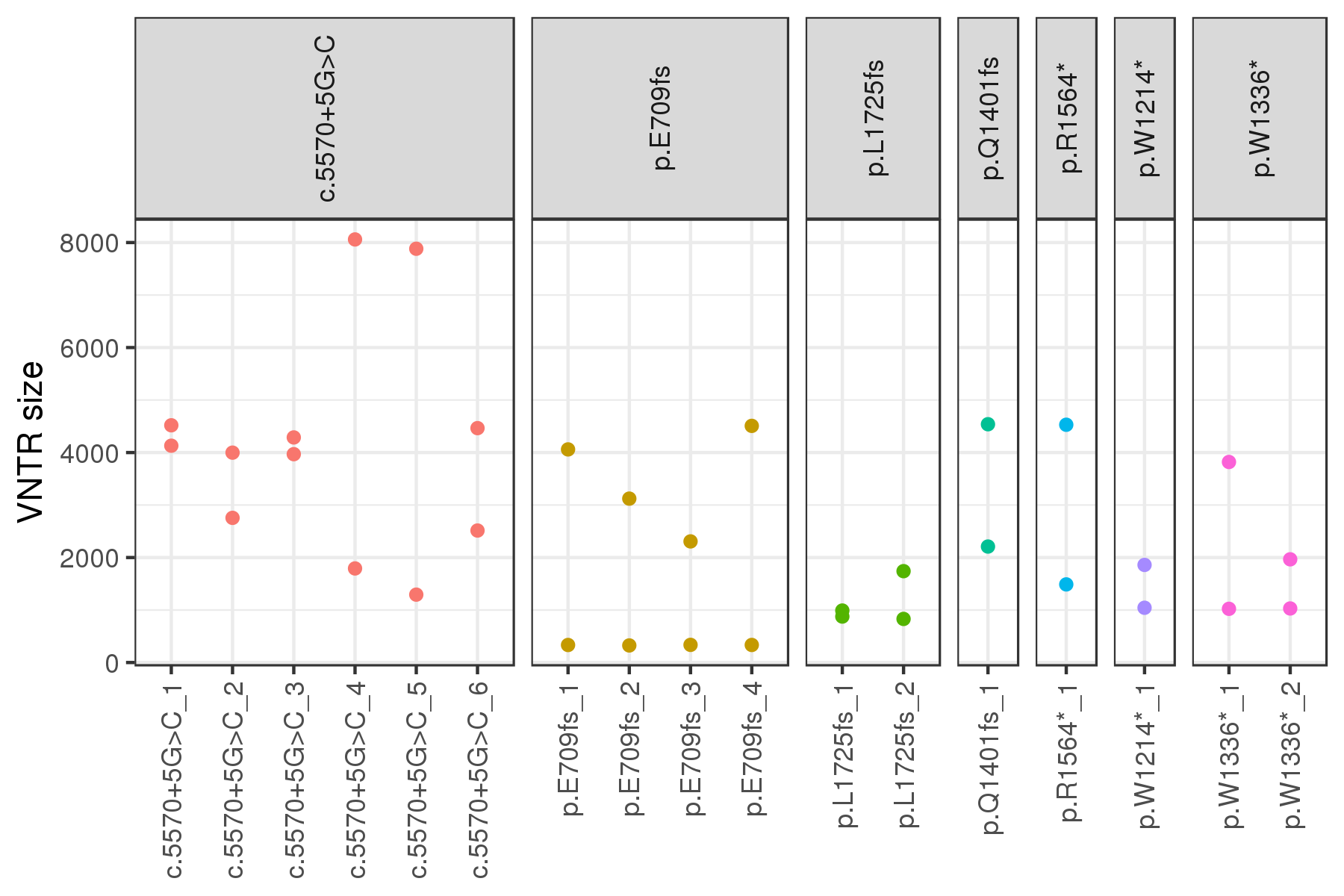


Southern blotted VNTR sizes in individuals carrying an *ABCA7* PTC mutation. Each panel groups the individuals (labels on the bottom), carrying the same PTC mutation (denoted on top). Two individuals (c.5570+5G>C_4 and c.5570+5G>C_5) carry an expanded VNTR allele (>5720bp), though no consistent haplotype sharing between expanded VNTR alleles and c.5570+5G>C is observed.

**Fig. S8: Allele-specific expression**

**
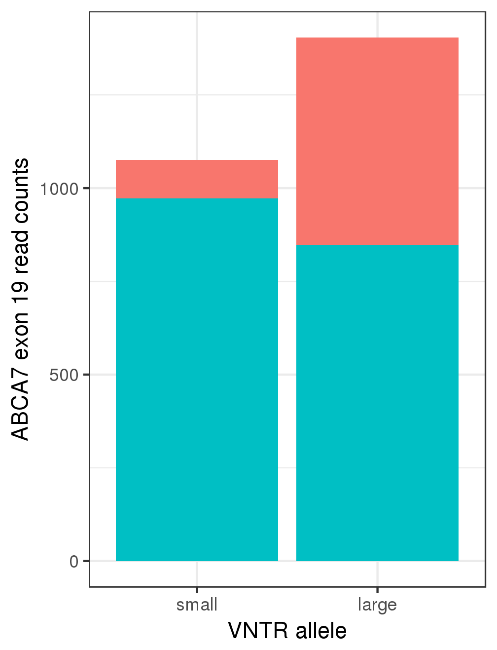
**

Allele-specific expression for an individual with a small wild-type (837 bp) and expanded (9711 bp) VNTR allele. The abundance of sequencing reads (y-axis) that are canonically spliced from exon 19 to exon 20 (blue) and reads that show exon 19 skipping (red) is shown for the small and large allele (x-axis), as determined by rs3752240 genotype.

**Fig. S9: Cycloheximide treatment**


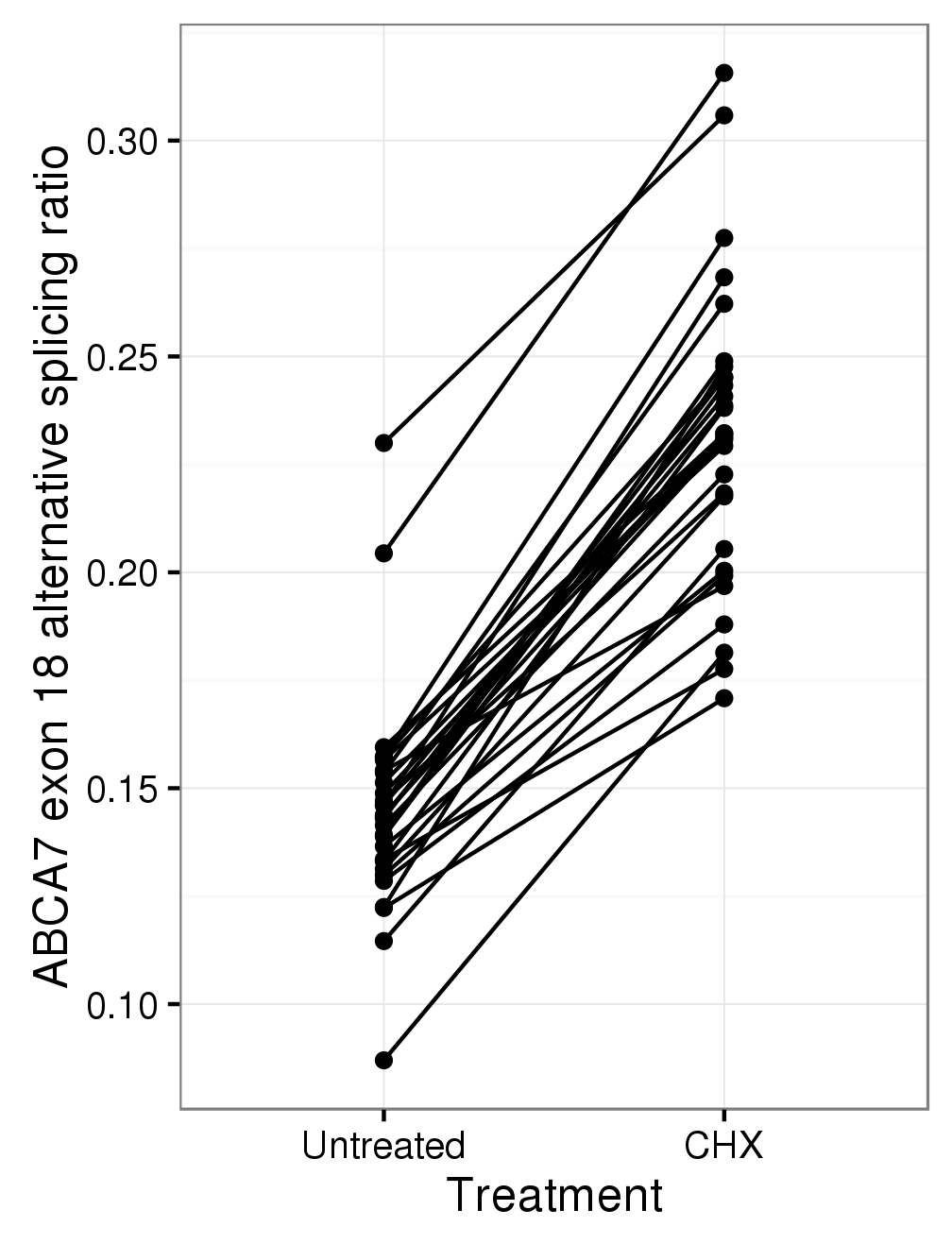


The exon 18 alternative splicing ratio (y-axis) is shown for LCL grown under normal conditions (Untreated), or LCL supplemented with cycloheximide (CHX). The same individuals (dots) are connected with a line.

**Fig. S10: Fold change after cycloheximide treatment**


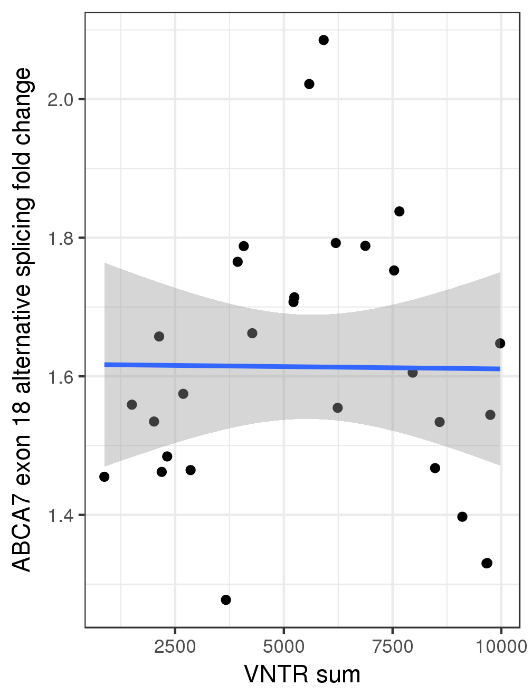


The exon 18 alternative splicing fold change (y-axis) for individuals (dots) between Untreated and CHX LCL growth as shown in Fig S9 is shown in relation to the sum of VNTR alleles (x-axis). A linear trend line (blue) is shown with standard error (grey).

# Supplementary tables

**Table S1: Overall study population**

|  | **AD patients** | | | **Controls** | | |
| --- | --- | --- | --- | --- | --- | --- |
| **Study population** | **n (%)** | **AAO (SD)** | **% female** | **n (%)** | **AAI (SD)** | **% female** |
| NGS | 772 (50%) | 74.6 (8.9) | 65% | 757 (50%) | 73.9 (9.0) | 60% |
| Southern Blotting | 275 (61%) | 74.1 (9.5) | 64% | 177 (39%) | 74.3 (9.8) | 61% |
| LCL | 21 (51%) | 73.4 (8.3) | 52% | 20 (49%) | 74.5 (6.5) | 65% |
| CSF | 168 (100%) | 76.6 (7.9) | 64% | - | - | - |

Summary statistics for BELNEU individuals according to the experiments in which they were included. Lymphoblastoid cell line (LCL) and cerebrospinal fluid (CSF) cohorts are subgroups of the Southern blotting cohort. NGS = individuals with next generation sequencing data available for VNTR coverage analysis, AAO = age at onset, AAI = age at inclusion.

**Table S2: Expanded *ABCA7* VNTR allele carriers**

|  |  |  |  |  |  | ***ABCA7* VNTR allele** | |
| --- | --- | --- | --- | --- | --- | --- | --- |
| **Phenotype** | **AAO/AAI** | **Familial history** | **Gender** | ***APOE*** | **rs3764650** | **Large (bp)** | **Small (bp)** |
| AD | 44 | NA | f | ε3ε3 | TT | 7303 | 1030 |
| AD | 46 | F | f | ε3ε3 | NA | 6243 | 4835 |
| AD | 60 | F | f | ε4ε4 | GT | 6383 | 3830 |
| AD | 65 | NA | f | ε3ε3 | TT | 9217 | 2447 |
| AD | 66 | S | m | ε3ε3 | GG | 8650 | 2786 |
| AD | 69 | S | f | ε4ε4 | GT | 7992 | 1202 |
| AD | 74 | S | f | ε3ε4 | GT | 8319 | 2400 |
| AD | 75 | F | m | ε3ε4 | GT | 6327 | 3168 |
| AD | 76 | F | f | ε3ε4 | TT | 6235 | 1808 |
| AD | 77 | S | f | ε3ε4 | GT | 5761 | 2901 |
| AD | 79 | F | f | ε3ε3 | GT | 6088 | 3187 |
| AD* | 79 | S | f | ε3ε3 | TT | 9711 | 837 |
| AD | 80 | S | m | ε3ε3 | TT | 6387 | 3768 |
| AD | 80 | S | m | ε3ε4 | GT | 7296 | 2682 |
| AD | 80 | S | f | ε3ε3 | GT | 5783 | 2064 |
| AD | 83 | S | f | ε3ε4 | GT | 7137 | 4664 |
| AD | 84 | S | f | ε3ε4 | TT | 7549 | 4586 |
| AD | 85 | S | f | ε3ε3 | GT | 9555 | 1756 |
| AD | 90 | S | m | ε3ε4 | TT | 5747 | 2724 |
| AD | NA | S | f | ε3ε4 | GT | 6063 | 2337 |
| CON | 63 | - | f | ε3ε4 | GT | 9054 | 444 |
| CON | 74 | - | f | ε3ε4 | GT | 10678 | 1718 |
| CON | 79 | - | m | ε3ε3 | GT | 7227 | 840 |

Summary of all individuals carrying an expanded *ABCA7* VNTR allele (> 5720 bp). AD = Alzheimer’s disease patient, CON = Healthy elderly control, AAO/AAI = Age at onset for patients, and age at inclusion for controls, NA = not available, F = positive familial history, S = sporadic, f = female, m = male, *APOE* = *APOE* genotype, rs3764650 = genotype for this AD-associated *ABCA7* GWAS SNP (T = ancestral allele, G = risk increasing allele). For each individual the corresponding largest and smallest VNTR alleles are shown in basepairs (bp). * LCL from this individual were used for allele-specific expression.

**Table S3: Linear regression results based on VNTR sum and the largest VNTR allele**

|  | VNTR sum | | | Largest VNTR allele | | |
| --- | --- | --- | --- | --- | --- | --- |
| Dependent variable | β | s.e. | p-value | β | s.e. | p-value |
| Overall ABCA7 expression | -4.7E-05 | 1.9E-05 | 1.3E-02 | -9.7E-05 | 3.5E-05 | 5.4E-03 |
| exon 19 skipping | 1.4E-05 | 2.2E-06 | 3.2E-13 | 2.5E-05 | 4.3E-06 | 1.2E-12 |
| Exon 18 alternative | 1.0E-06 | 1.7E-06 | 5.0E-01 | 1.5E-06 | 3.2E-06 | 4.2E-01 |
| Intron 18 retention | 5.1E-07 | 5.7E-07 | 3.5E-01 | 1.0E-06 | 1.1E-06 | 3.2E-01 |
| Amyloid β CSF | -4.6E-05 | 2.1E-05 | 2.6E-02 | -5.3E-05 | 2.9E-05 | 6.6E-02 |
| P-tau CSF | 4.0E-05 | 2.3E-05 | 9.2E-02 | 4.0E-05 | 3.2E-05 | 2.2E-01 |
| T-tau CSF | 2.1E-05 | 3.0E-05 | 4.8E-01 | 1.2E-05 | 4.2E-05 | 7.8E-01 |
| Age at onset | 2.7E-06 | 2.1E-04 | 9.9E-01 | 5.9E-05 | 3.1E-04 | 8.5E-01 |

The β regression coefficients, standard errors (s.e.) and p-values are shown for all regression analyses conducted with either the sum of VNTR alleles, or the largest VNTR allele as the independent variable.

**Table S4: Association with age at onset corrected for gender and *APOE***

| Independent variable | β | s.e. | p-value |
| --- | --- | --- | --- |
| VNTR sum | 2.7E-06 | 2.1E-04 | 9.9E-01 |
| gender (male) | -1.0E+00 | 9.4E-01 | 2.9E-01 |
| *APOE* | -1.8E+00 | 7.3E-01 | 1.3E-02 |

The β regression coefficients, standard errors (s.e.) and p-values are shown for the regression model: Age at onset (AAO) ~ VNTR sum + gender + *APOE* (with *APOE* noted as 0,1, or 2 depending on the number of ε4 alleles).

# Supplementary references

1. 1000 Genomes Project Consortium, Auton A, Brooks LD, Durbin RM, Garrison EP, Kang HM, et al. (2015) A global reference for human genetic variation. Nature 526:68–74. doi: 10.1038/nature15393

2. Cuyvers E, De Roeck A, Van den Bossche T, Van Cauwenberghe C, Bettens K, Vermeulen S, et al. (2015) Mutations in ABCA7 in a Belgian cohort of Alzheimer’s disease patients: a targeted resequencing study. Lancet Neurol 14:814–822. doi: 10.1016/S1474-4422(15)00133-7
